# *Hellwigia opalina* (Zingiberaceae) – a new species of the enigmatic jade gingers of Sulawesi

**DOI:** 10.3897/phytokeys.272.171221

**Published:** 2026-04-03

**Authors:** Axel Dalberg Poulsen, Seni Kurnia Senjaya, Marlina Ardiyani, Mark Fleming Newman

**Affiliations:** 1 Royal Botanic Garden Edinburgh, Edinburgh EH3 5LR, Scotland, UK Royal Botanic Garden Edinburgh Edinburgh United Kingdom; 2 Department of Botany, University of British Columbia, Vancouver, BC, Canada University of British Columbia Vancouver Canada; 3 Research Center for Biosystematics and Evolution, Badan Riset dan Inovasi Nasional (BRIN), Bogor, West Java, Indonesia Research Center for Biosystematics and Evolution, Badan Riset dan Inovasi Nasional (BRIN) Bogor Indonesia

**Keywords:** *
Alpinia
*, *ex situ* conservation, Indonesia, monoecism, Wallacea

## Abstract

The Indonesian Island of Sulawesi is home to a number of members of the ginger family, Zingiberaceae, with unusual turquoise flowers not found anywhere else. A recent paper reinstating *Hellwigia*, using molecular evidence, placed these “jade gingers” in this genus. During a joint expedition between the Royal Botanic Garden Edinburgh and Bogor Botanical Gardens 26 years ago, an interesting jade ginger was collected at Mt. Sojol. This is similar to two other named species from Sulawesi, *H. coeruleoviridis* and *H. glacicaerulea*, in having unusual dimorphic, turquoise-bluish green flowers but differs from both in the much shorter ligule without a demarcation line and is described here as *Hellwigia opalina*. In preparation for this, all available material of jade gingers from Sulawesi was examined, and all except the collections from Mt. Sojol had a long ligule with a demarcation line similar to *H. coeruleoviridis* and *H. glacicaerulea*. It is, however, questionable whether the identification by R.M. Smith of several collections from Lore Lindu National Park, Central Sulawesi, matches the type of *H. coeruleoviridis*, which was lost in Berlin during the Second World War. Until this species has been recollected at its type locality, here identified as Mt. Tentolomatinan, 300 km away, the identification cannot be easily ascertained. Rhizomes from Mt. Sojol were cultivated in botanic gardens, and plants cultivated at the Royal Botanic Garden flowered several times, which enabled detailed studies and dissection of both flower morphs. This is usually impossible during fieldwork and emphasizes the importance of cultivation of gingers—not just for *ex situ* conservation but also for research purposes. Detailed photographs taken of the cultivated plants were used to obtain a better morphological understanding and to illustrate *Hellwigia opalina* in the present paper.

## Introduction

Within the last 25 years, several studies involving molecular data (Rangsiruji et al. 2000; Kress et al. 2002; De Boer et al. 2018; Docot 2025) have found that *Alpinia* Roxb. is polyphyletic. Kress et al. (2005, 2007) referred to six clades within the Alpinioideae: Carolinensis, Eubractea, Fax, Galanga, Rafflesiana, and Zerumbet. As taxonomy should ideally reflect evolution (Zaman et al. 2025), only the Galanga clade should remain as *Alpinia*, as it includes the type, *A. galanga* (L.) Willd. After including samples of *Elettaria cardamomum* (L.) Maton (the type of the genus), De Boer et al. (2018) and Poulsen et al. (2018) found that this species is placed within the Fax clade, and Poulsen et al. (2024) recircumscribed *Elettaria* to include seven species. In 2025, Senjaya et al. reinstated *Hellwigia* Warb. as the name for the Carolinensis clade, as the type of the genus, *H. pulchra* Warb., is the oldest name placed in this group. While the Eubractea clade is currently being revised by Docot et al. (in prep.), recircumscriptions of the Rafflesiana and Zerumbet clades are still pending.

Sulawesi is a large island east of Borneo in Wallacea. The Zingiberaceae of Sulawesi are poorly known, the latest account of them being Schumann’s monograph of the family in “Das Pflanzenreich” (Schumann 1904). Very little plant collecting has been conducted in Sulawesi. The most recent estimate is around 23 specimens per 100 km^2^ (Whitten 1987), probably the lowest collecting intensity of any Indonesian island. Subsequently, exploration of gingers has, however, increased by Poulsen (2012), Ardiyani et al. (2020, 2021), and other Indonesian botanists.

The Royal Botanic Garden Edinburgh and Bogor Botanical Gardens (Kebun Raya Indonesia) conducted three joint botanical expeditions in Sulawesi in 2000, 2002, and 2004. Each time, the Zingiberaceae were among the plant families targeted. During the expedition of 2000, the collecting team visited Mt. Sojol, a high mountain in the northern part of Central Sulawesi Province that has rarely been visited by botanists. Certain species of Zingiberaceae were collected in flower for the herbarium, whereas others were either sterile or only in fruit and were also collected for cultivation. One of these accessions subsequently flowered for several years in a glasshouse in Edinburgh, bearing striking turquoise (bluish green) flowers. In the present paper, we simply refer to this as a “jade ginger.”

Here, we present evidence for the inclusion of this species as a new member of the genus *Hellwigia* and provide additional information about other species of jade gingers in Sulawesi. Having the plant in cultivation allowed for detailed studies of the floral morphology, phenology, and other aspects requiring long-term observation. Key characters of the three jade ginger species are compared, along with Smith’s identification of specimens from Lore Lindu NP.

## Material and methods

Fieldwork was carried out following standard collecting methods (Bridson and Forman 1992) and with all necessary permits. The first set of herbarium collections from the expedition, including Mt. Sojol, was deposited at BO, and duplicates were exported with permission. Descriptions and measurements were based on a combination of characters observed in the field (mainly colors) as well as on specimens, in which the flowers are known to be turquoise-blue, from the following herbaria: BO, CEB, E, L, and SING (herbarium codes follow Thiers, continuously updated). Morphological terminology mostly follows Smith (1990, 1991) and Beentje (2010), while Hewson (1988) was used to describe the indumentum. Conservation status was assessed according to the IUCN Red List Categories and Criteria (IUCN 2024). A distribution map was made using geographical coordinates extracted from herbarium specimen labels or by matching the travel route of the Sarasin cousins (Sarasin and Sarasin 1905) with present-day maps. The description of the new species in the present paper was largely based on a living collection producing both functionally female (pistillate) and functionally male (staminate) flowers. The former was described from spirit material of *M.F. Newman & J. Leong-Škorničková* 1459 (E). The functionally male flower was described from spirit material of *M.F. Newman* 2537 (E). This was repeated from material of both types of flowers of *A.D. Poulsen* 3255 (E).

## Results

Comparison of the material from Mt. Sojol with published descriptions of jade gingers and herbarium material did not lead to a conclusive identification. Senjaya et al. (2025) sequenced a sample of this material using the nuclear ribosomal internal transcribed spacer (ITS) region and placed it in *Hellwigia* (GenBank acc. no. PV158161). This is also supported morphologically by the dimorphic flowers, which are arranged in cincinni and not evenly distributed along the rachis. The short ligule without a demarcation line, however, is unlike that of any other collection or known species of jade ginger. We therefore describe the new species below and discuss the challenges of identifying other jade gingers in Sulawesi.

**Figure 1.**
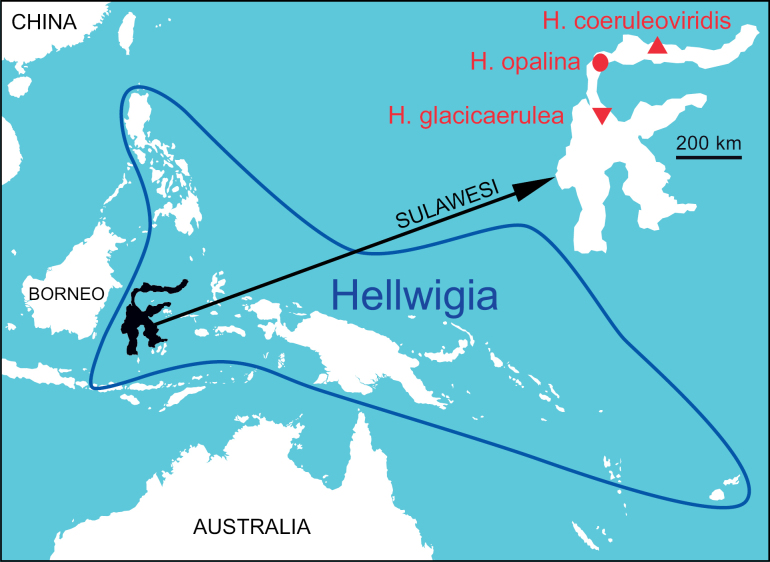
Approximate distribution of *Hellwigia* east of Huxley’s Line. Close-up of the Indonesian island of Sulawesi indicating the type localities of the three jade ginger species. Map by A.D. Poulsen.

*Hellwigia* in its current circumscription comprises at least 76 species distributed east of Huxley’s Line (including Wallace’s Line), through Wallacea and New Guinea into the Western Pacific, but excluding Australia (Senjaya et al. 2025; Fig. 1). The new species increases the number of species in *Hellwigia* to 77 and the number of jade gingers to three, all of which are endemic to the island of Sulawesi.

### Taxonomic treatment

#### Hellwigia opalina

Taxon classificationPlantaeHymenopteraIchneumonidae

Ardiyani & A.D.Poulsen, sp. nov.

52D54EB3-0D44-5BD8-BE9E-B556EC14D694

urn:lsid:ipni.org:names:77378263-1

Figs 2, 3

##### Diagnosis.

Similar to *Hellwigia glacicaerulea* (R.M.Sm.) Senjaya & A.D.Poulsen in having inflorescences of secund cincinni bearing turquoise-blue, dimorphic flowers but differing in its shorter ligule (2–3.5 mm, coriaceous vs. 20–30 mm, marcescent), shorter petiole (7–12 mm vs. 20–30 mm), leaf blades being densely tomentose beneath (vs. shortly tomentose), and tomentose inflorescence rhachis (vs. shortly tomentose).

**Figure 2.**
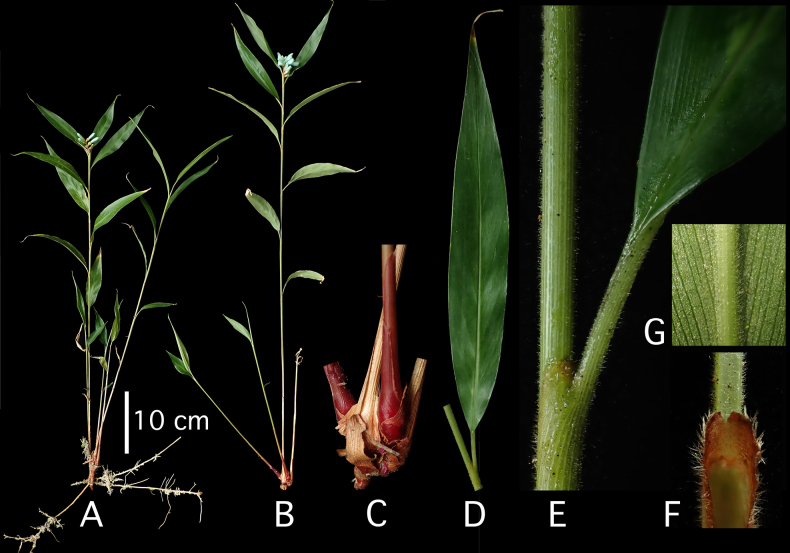
*Hellwigia opalina* Ardiyani & A.D.Poulsen, sp. nov. **A**. Leafy shoots, inflorescence with functionally female flowers open; **B**. Leafy shoots, inflorescence with functionally male flowers open; **C**. Bases of leafy shoots; **D**. Part of pseudostem with one leaf blade; **E**. Close-up of ligule, petiole, and leaf base; **F**. Ligule, viewed from the inside; **G**. Close-up of midrib beneath. All photographs of the type (*A.D. Poulsen* 3255) by A.D. Poulsen.

**Figure 3.**
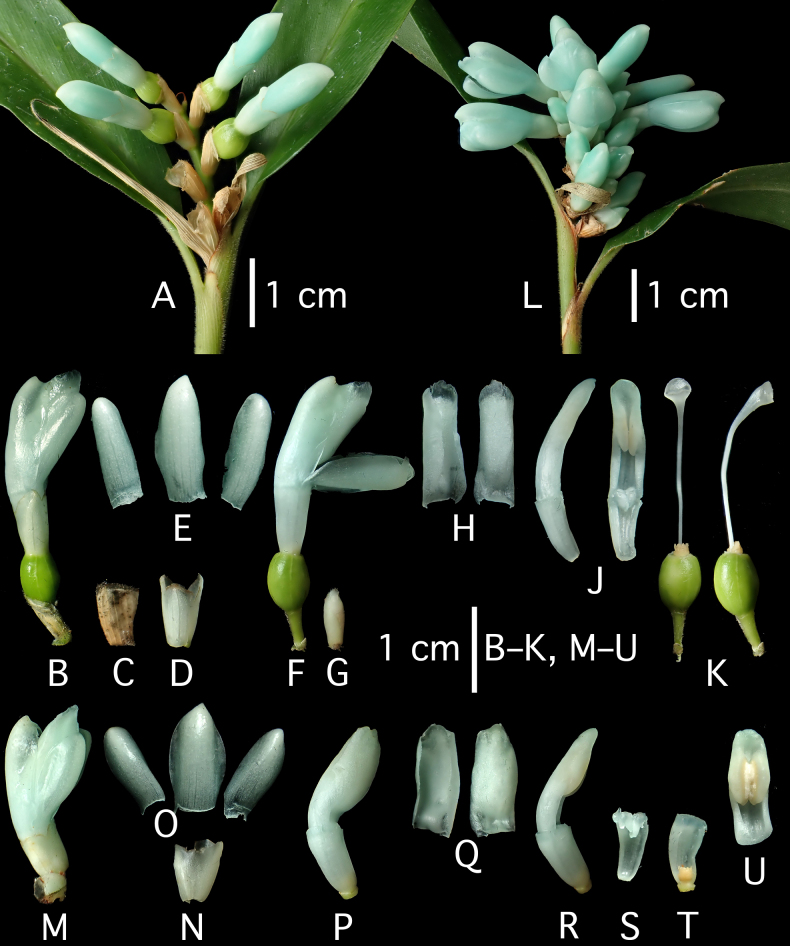
*Hellwigia opalina* Ardiyani & A.D.Poulsen, sp. nov. Functionally female flowers: **A**. Inflorescence with first flowers; **B**. Flower; **C**. Bracteole; **D**. Calyx; **E**. Corolla lobes; **F**. Flower, bracteole removed; **G**. Inner part of cincinnus with functionally male flowers in bud; **H**. Labellum, ventral and dorsal view; **J**. Stamen, lateral and ventral view (staminodes); **K**. Gynoecium, ventral and lateral view. Functionally male flowers: **L**. Inflorescence; **M**. Flower; **N**. Calyx; **O**. Corolla lobes; **P**. Flower, bracteole removed; **Q**. Labellum, ventral and dorsal view; **R**. Floral tube and stamen, lateral view; **S**. Floral tube and staminodes; **T**. Floral tube and epigynous gland; **U**. Stamen, ventral view. All photographs of the type (*A.D. Poulsen* 3255) by A.D. Poulsen.

##### Type.

Cultivated at the Royal Botanic Garden Edinburgh, Accession Number 20000477*E, G57 Tropical Montane House, 15 August 2022, *Poulsen* 3255 (holo BO; iso CEB, E [E01530574–5], L, SING). The cultivated plant was grown from rhizomes collected on 26 February 2000, *P. Smith & L. Galloway* 82, at the same locality and collection date as *Argent & al*. 00175 (see below): Indonesia • Central Sulawesi, Mt. Sojol, c. 0°40'N, 120°10'E, c. 1500 m elev.

##### Description.

Terrestrial perennial herb in dense clump. ***Leafy shoots*** with up to 13 blades distributed in upper half; pseudostem 60–90 cm long; base to 1.3 cm across, reddish brown; lower sheaths turning pale brown and dry, upper pale mid-green, faintly striate, scattered tomentose, denser near ligule; ligule 2–3.5 mm long, entire (sometimes splitting with age), pubescent, especially towards margin, brownish; petiole 7–12 mm long, canaliculate, tomentose, pale mid-green; lamina narrowly ovate, 14–23 × 2.5–3.4 cm, glabrescent or scattered tomentose above, densely tomentose on midrib and lamina beneath, dark green above, pale beneath, base cuneate to obtuse, margin tomentose near apex, tapering towards apex. ***Inflorescence*** terminal to the leafy shoot, erect, unbranched, rhachis shortly tomentose; inflorescence bracts 1–2, 3.5–6 cm long, drying pale brown; flowering part to 6 cm long; rhachis 3.5–4.5 cm long, tomentose; cincinni 14–18, alternate and distichous (secund), congested in upper half of rhachis, cincinnus bracts absent, cincinnus bearing 4–6 flowers, the first functionally female and subsequent flowers functionally male; bracteoles broadly funnel-shaped, telescoping, to 8 mm long at most on proximal cincinni, reducing in size along cincinni and on distal cincinni, deeply split down one side, tomentose, ciliate at margin, faintly greenish white to pale bluish, becoming dry, pale brown, and brittle, lacerating somewhat at anthesis. ***Flowers*** pale turquoise-blue. ***Functionally male flowers*** c. 20 mm long (excl. pedicel), pedicel 2–5 mm long, pubescent; calyx to 6 mm long, slightly irregularly split at apex; floral tube to 8 mm long, glabrous; dorsal corolla lobe 11–13 × 9 mm (when flattened), cucullate, apex swollen; lateral corolla lobes 10–11 × 7 mm (when flattened), asymmetrical, cucullate, flatter than dorsal one, margin slightly lacerate; labellum 10–12 × 5 mm (when flattened 8 mm wide, ovate), spoon- to boat-shaped, thickly fleshy in center, margin membranous, inrolled, apex rounded; lateral staminodes meeting across inside of corolla tube; stamen 10 × 4 mm, sparsely pubescent on abaxial surface, filament 3–3.5 mm long, canaliculate, anther to 8 × 4 mm, base spurred, sparsely tomentose adaxially, thecae opening by longitudinal slits for c. 4.5 mm from 0.5 mm above base, full of pollen, anther crest 2–3 mm long (above thecae), rounded, margin sparsely ciliate; ovary < 1 mm long, empty; epigynous glands 1.5 mm long, at abaxial side of base of style, irregularly fleshy, pale yellow; style and stigma to 4 mm long, not fully developed. ***Functionally female flowers*** c. 25 mm long (excl. pedicel), pedicel to 4 mm long when flowering, extending to 7 mm on fruits, sericeous; calyx 8 mm long, tubular, unevenly trilobed, glabrous, white tinged slightly blue; floral tube 3–7 mm long, glabrous; dorsal corolla lobe 10–13 × 7–8 mm (when flattened), cucullate, ± tomentose at apex, apex swollen; lateral corolla lobes 7–13 × 5.5–7 mm (when flattened), asymmetrical, flatter than dorsal one, cucullate, margin slightly lacerate, apex swollen; labellum 9.5–12 × 5 mm, (when flattened 9 mm wide, ovate), boat-shaped, thickly fleshy in center, margin membranous, inrolled, apex rounded; lateral staminodes 2.5 mm long, fleshy, deflexed; stamen 9–12 × 3 mm, fleshy, filament c. 3 mm long, canaliculate, anther 7–8 × 3.5 mm, base spurred, sparsely tomentose adaxially, crest 1–1.5 mm long, rounded, margin sparsely ciliate, thecae ± parallel, empty; ovary 5–7 × 5–5.5 mm, ovoid to ellipsoid with faint longitudinal ridges, trilocular with parietal placentation and many ovules, glabrous, with very few erect hairs at base, shiny, yellow-green; epigynous glands 1.5 mm long, at abaxial side of base of style, irregularly fleshy, pale yellow; style to 16 mm long, white; stigma 2 × 3 mm, club-shaped, ostiole in center abaxially, transverse 2.5 mm, margin ciliate. ***Fruit*** immature, globose to ellipsoid, 6 × 4–5 mm, pale yellow (mature fruit expected to be at least twice as large); seeds not seen.

##### Distribution and habitat.

Indonesia, Central Sulawesi, Mt. Sojol, 1500 m elevation. Known only from the type locality and cultivated material from the same population (Fig. 1). This species grows in submontane ridge forest.

##### Etymology.

The epithet means ‘opal-colored’ from Greek, opallios, an opal.

##### Conservation status and preliminary IUCN Red List assessment.

Only one population has been sampled at Mt. Sojol (*Argent et al*. 00175; *Smith & Galloway* 82), and, as the area is protected as a Reserve, the species may therefore be considered as LC (IUCN 2019).

##### Additional specimens examined

**(*paratypes*)**. Indonesia • Central Sulawesi Province. Mt. Sojol; c. 0°40'N, 120°10'E; c. 1500 m elev.; 26 February 2000; *G.C.G. Argent, C.M. Mendum & Hendrian* 00175 (BO n.v., E [E00103765–66], L [QR code L.4195953]) • from same locality, cultivated RBGE, Acc. No. 20000477, no qualifier (origin as previous), G66; 30 April 2006; *M.F. Newman & J. Leong-Škorničková* 1459 (E [E00228081; spirit E00830219]) • Acc. No. 20000477*E, G57 Tropical Montane House; 29 August 2012; *M.F. Newman* 2537 (E [E01378134; spirit E00412407]).

##### Notes.

*Hellwigia opalina* is part of a group of closely related, slender, small-leaved species formerly placed in *Alpinia* section *Myriocrater* K.Schum. These species are distinct from the typically robust members of the section and are notable for their icy blue or turquoise flowers. The other species in the group are *H. coeruleoviridis* (K.Schum.) Senjaya & A.D.Poulsen and *H. glacicaerulea* (R.M.Sm.) Senjaya & A.D.Poulsen. *Hellwigia opalina* can most easily be distinguished from both species by its much shorter ligule (2 mm vs. 20–45 mm).

Monoecism is a common sexual system in *Alpinia* section *Myriocrater*, in which the jade gingers of Sulawesi were placed (Burtt and Smith 1972; Smith 1977). The first and, occasionally, the second flower of each cincinnus is functionally female, while the remaining flowers are functionally male (Burtt and Smith 1972). Cultivating the plants allowed us to study both kinds of flowers, leading to a more detailed description of this species.

### Identification of other species of jade ginger of Sulawesi

Including the present paper, three species of jade ginger have been published, all from the Indonesian island of Sulawesi: *Hellwigia coeruleoviridis*, *H. glacicaerulea*, and *H. opalina* (Fig. 1). Most collections were made at Mt. Nokilalaki or Mt. Rore Katimbu, both mountains in Lore Lindu NP, Central Sulawesi. Smith (1991) thought that two species occurred at Lore Lindu. The first one she identified as *Alpinia coeruleoviridis* K.Schum. (focusing on *J. Johansson et al*. 240), and the second, *A. glacicaerulea* R.M.Sm., she described as new but, in the diagnosis, only mentioned a difference in leaf shape (elliptic vs. narrowly ovate; Table 1). We have studied a total of 10 collections (see list below) from Lore Lindu NP, including what Smith (and M.F. Newman) identified as *Alpinia coeruleoviridis* and the type of *A. glacicaerulea*, *M. van Balgooy* 3194 (E, L), and find that there is a continuum in leaf shape from elliptic to narrowly ovate. All seem more or less stilt-rooted and probably represent a single species. More detailed studies of floral morphology using fresh or pickled material, as well as population genetics, will help to clarify this.

**Table 1.** A comparison of key characters among the three jade ginger species, including Smith’s identification of collections from Lore Lindu NP. The information is based on: *H. coeruleoviridis* (Schumann 1899, 1904, including characters in the species identification keys); *H. coeruleoviridis* sensu Smith (Smith 1991; especially *Johansson* et al. 240, which she cited, but also other collections from the same area of the same taxon); *H. glacicaerulea* (Smith 1991; *van Balgooy* 3194, the type); *H. opalina* (*Poulsen* 3255, the type). Major differences are shown in bold.

Character	*H. coeruleoviridis*	*H. coeruleoviridis* sensu Smith	*H. glacicaerulea*	*H. opalina*
Height (cm)	20 to barely 30	70–150	to c. 100	60–90
Ligule length (mm)	To 25	37–45	20–30	2–3.5
Ligule structure	Membranous	Membranous, marcescent	Membranous, marcescent	Coriaceous
Petiole length (mm)	To 20	26–30	20–30	7–12
Lamina shape	Linear to narrowly ovate	Narrowly ovate	Elliptic	Narrowly ovate
Lamina dimension (cm)	To 14 × 2	19–36 × 3.1–4.5	8–10 × 4 [type 13 × 4.5]	14–23 × 2.5–3.4
Lamina indumentum	Minutely hairy, otherwise glabrous on both sides	Sparsely tomentose on both sides (rarely glabrous above)	Shortly tomentose on both sides (type), not glabrous as stated in protologue	Glabrescent above, densely tomentose beneath
Inflorescence	Erect rarely pendulous	Erect	Erect	Erect
Inflorescence bracts length (cm)	To 3, often much smaller	To 8, often missing, leaving a scar	Unknown	3.5–6
Inflorescence peduncle (cm)	Short or absent	0–3	0–6 cm	Absent
Length of inflorescence flowering part (cm)	Barely 2	5–12	3–5	To 6
Rhachis indumentum	Glabrous	Glabrous or sparsely ciliate	Sparsely tomentose (type)	Tomentose
Cincinni arrangement	Sessile, conspicuously distant from each other, spreading	Sessile, ± congested, secund	Sessile, tightly congested, secund	Sessile, congested in upper half of rhachis, secund
Number of cincinni	5	7–25	7–9	14–18
Flowers, color	Blue-green	Pale bluish green	Bluish	Pale turquoise-green
Functionally male flower:				
Calyx length (mm)	9	7	7–8	To 6
Floral tube length (mm)	16	c. 5	Not informed	To 8
Dorsal corolla lobe length (mm)	To 20	10	To 10	11–13
Lateral corolla lobe length (mm)	To 17	10	To 10	10–11
Labellum length (mm)	To 20	12	10	10–12

The type of *H. coeruleoviridis* is assumed lost when the herbarium in Berlin was firebombed by Allied forces during the Second World War, and all that is known is what Schumann published (1899, 1904) in German and Latin, including an illustration of a whole flower. Despite Schumann describing this species as “the smallest of the entire genus [*Alpinia*], barely 30 cm tall,” Smith (1991) considered *Johansson et al*. 240 of a 1.4 m tall plant (one of the 10 collections mentioned above) from 300 km away at Lore Lindu NP as conspecific. There are several other differences, such as the much lower number of cincinni, not being secund, and surprisingly much larger corolla lobes and labellum (Table 1). It is possible that the Sarasin cousins collected the now-lost type of *H. coeruleoviridis* at a ridge-top habitat where the plants may have been unusually small, but that would not explain the larger flowers. Until the species has been collected again at the type locality and the variation of characters better understood, it is too early to say if this name should be applied to any material in Lore Lindu NP. Considering, however, the several differences between Schumann’s description and collections at Lore Lindu NP, it seems likely that *H. coeruleoviridis* does not occur at Lore Lindu NP and that there are at least three distinct, named species of jade gingers in Sulawesi.

Disregarding the exact identity and number of species, examination of all appropriate collections of *Hellwigia* from Sulawesi (see list of material below) reveals that they are relatively small plants, less than 1 m (rarely 1.5 m), growing at altitudes of 1500–2200 m in the provinces of Gorontalo, Central Sulawesi, South Sulawesi, and West Sulawesi.

Turquoise, bluish green flowers are rare among the angiosperms. The most iconic example is the jade vine, *Strongylodon macrobotrys* A.Gray (Fabaceae), endemic to the Philippines. Despite the second author having focused on sexual systems and pollination of *Hellwigia* species during fieldwork in Central Sulawesi, we have no observations or documentation of the pollinators yet. Beetles were seen visiting jade ginger plants, but further evidence is needed to confirm whether they play a pollinating role. Meanwhile, the unusual flower color makes jade gingers attractive potential ornamentals.

The fruits are globose to ellipsoid, probably up to about 1.5 cm in diam., and orange-red when mature. Even though we have not seen mature fruits of any of the types, in all other jade ginger collections examined, the seeds are not enclosed by an aril. The seed disperser also remains a mystery, but given the color, one may hypothesize that it may be birds.

Senjaya et al. (2025) included four samples of jade gingers in a phylogenetic analysis using ITS. The resolution within *Hellwigia* is generally poor, and, without a more advanced approach, it is too early to say if jade gingers form a monophyletic clade within the genus, but at this stage it does not seem to be the case.

### Other jade ginger collections considered or examined

*H. coeruleoviridis*. Indonesia • Sulawesi. Border between Central and Gorontalo Provinces, Matinang Mountain Chain [Mount (Huidu) Tentolomatinan], estimated at 1400–2000 m elev., flowering 28 August 1894, *P.B. & K.F. Sarasin* 649 (holotype B, assumed lost during the Second World War). Flower illustrated in Schumann (1904; fig. 42H). *H. coeruleoviridis* (sensu Smith, cf. 1991: fig. 1B, Lore Lindu): Indonesia. Central Sulawesi Province • Lore Lindu National Park: Mt. Nokilalaki, montane forest; 1°13'5"S, 120°9'0"E; 1475 m elev.; 1 March 2008; *A.D. Poulsen & Firdaus* 2652 (AAU, BO [Sheet No. BO-1882762], E [E00320613; spirit E00412043–44]) • same loc.; 1°14'35"S, 120°9'8"E; 1875 m elev.; 3 March 2008; *A.D. Poulsen & Firdaus* 2659 (AAU, BO [Sheet No. BO-1882761], E [E00318727–29], no spirit!) • same loc.; 1°14'35"S, 120°9'8"E; 1875 m elev.; 3 March 2008; *A.D. Poulsen & Firdaus* 2664 (AAU, BO [Sheet No. BO-1883949], E [E00318723–24]) • humid and foggy mountain forest; 1°16'59"S, 120°9'41"E; 2150 m elev.; 6 March 2008; *D. Cicuzza* 826 (BO n.v., CEB n.v., Berkeley n.v., L n.v., E [E00315772]) • same loc. and date; *D. Cicuzza* 810 (BO n.v., CEB n.v., Berkeley n.v., L n.v., E [E00311417]) • same loc., north slope, primary forest; 1°10'S, 120°10'E; 1700 m elev.; 9 March 1981; *J. Johansson, H. Nybom & S. Riebe* 240 (BO n.v., E [E00149601], L n.v.) • Mt Roroka Timbu, west slope, montane forest; 1°16'S, 120°18'E, 2050 m elev.; 16 May 1979; E.F. *de Vogel* 5410 (L [L 1468062]). *H. glacicaerulea*: Indonesia. Central Sulawesi Province • Mt Roroka Timbu [Rore Katimbu], W slope, montane forest dominated by *Agathis* 40 m tall; 0°30'–1°30'S, 119°30'–120°30'E; 2000 m elev.; 8 May 1979; *M. van Balgooy* 3194 (holotype L [barcode L 041069, QR code missing]; isotypes E [E00149606; spirit E00211716]) • same general loc.; 2100–2200 m elev.; 16 May 1979; *E. Hennipman* 5427 (L [barcode L 0427903/ QR code L 1480680]) • Mt. Nokilalaki; 1600 m elev.; 7 July 1939; *S. Bloembergen* 3897 (L [barcode L 0428645 / QR code L 1480678], BO [Sheet No. BO-1274694]54) • E of Lake Lindu, Mt. Nokilalaki; 1200–1500 m elev.; 30 April 1975; *W. Meijer* 9816 (L [barcode L 0427902 / QR code L 1480679]). *H.* sp.: Indonesia. South Sulawesi Province • NE of Karangan along trail towards Rantemario, montane forest, bryophytes abundant; >3°24'46.8"S, 120°0'3.6"E; 1900 m elev.; 28 January 2009; *A.D. Poulsen, Firdaus & Sahir Tiburrung* 2772 (BO, E [E00319303–04, E00412123 (spirit 4892)], SING). West Sulawesi Province • Mamasa, G. Gandang Dewata, primary forest on steep slope, 2°52'34.8"S, 119°23'8"E; 1905 m elev.; 26 April 2016; *M. Ardiyani, Anis, W. Wardani, P.K. Wardani & D. Wulansari* Sulbar068 (BO, 4 sheets).

## Supplementary Material

XML Treatment for Hellwigia opalina

## Additional information

### Use of AI

No use of AI was reported.

### Author ORCIDs

Axel Dalberg Poulsen https://orcid.org/0000-0002-7651-6439

Seni Kurnia Senjaya https://orcid.org/0000-0003-4952-1352

Marlina Ardiyani https://orcid.org/0000-0003-2453-3066

Mark Fleming Newman https://orcid.org/0000-0003-4851-014X

### Data availability

All of the data that support the findings of this study are available in the main text.
